# Mass Spectrometry Proteotyping-Based Detection and Identification of *Staphylococcus aureus*, *Escherichia coli*, and *Candida albicans* in Blood

**DOI:** 10.3389/fcimb.2021.634215

**Published:** 2021-07-26

**Authors:** Nahid Kondori, Amra Kurtovic, Beatriz Piñeiro-Iglesias, Francisco Salvà-Serra, Daniel Jaén-Luchoro, Björn Andersson, Gelio Alves, Aleksey Ogurtsov, Annika Thorsell, Johannes Fuchs, Timur Tunovic, Nina Kamenska, Anders Karlsson, Yi-Kuo Yu, Edward R. B. Moore, Roger Karlsson

**Affiliations:** ^1^ Department of Infectious Diseases, Sahlgrenska Academy, University of Gothenburg, Gothenburg, Sweden; ^2^ Department of Clinical Microbiology, Sahlgrenska University Hospital, Gothenburg, Sweden; ^3^ Culture Collection University of Gothenburg (CCUG), Sahlgrenska Academy of the University of Gothenburg, Gothenburg, Sweden; ^4^ Microbiology, Department of Biology, University of the Balearic Islands, Palma de Mallorca, Spain; ^5^ Bioinformatics Core Facility at Sahlgrenska Academy, University of Gothenburg, Gothenburg, Sweden; ^6^ National Center for Biotechnology Information (NCBI), Bethesda, MD, United States; ^7^ Proteomics Core Facility at Sahlgrenska Academy, University of Gothenburg, Gothenburg, Sweden; ^8^ Norra-Älvsborgs-Länssjukhus (NÄL), Trollhättan, Sweden; ^9^ Nanoxis Consulting AB, Gothenburg, Sweden

**Keywords:** blood-stream infections, proteotyping, MALDI-TOF MS, proteomics, bacteremia, fungemia, sepsis, rapid diagnostics of infectious diseases

## Abstract

Bloodstream infections (BSIs), the presence of microorganisms in blood, are potentially serious conditions that can quickly develop into sepsis and life-threatening situations. When assessing proper treatment, rapid diagnosis is the key; besides clinical judgement performed by attending physicians, supporting microbiological tests typically are performed, often requiring microbial isolation and culturing steps, which increases the time required for confirming positive cases of BSI. The additional waiting time forces physicians to prescribe broad-spectrum antibiotics and empirically based treatments, before determining the precise cause of the disease. Thus, alternative and more rapid cultivation-independent methods are needed to improve clinical diagnostics, supporting prompt and accurate treatment and reducing the development of antibiotic resistance. In this study, a culture-independent workflow for pathogen detection and identification in blood samples was developed, using peptide biomarkers and applying bottom-up proteomics analyses, i.e., so-called “proteotyping”. To demonstrate the feasibility of detection of blood infectious pathogens, using proteotyping, *Escherichia coli* and *Staphylococcus aureus* were included in the study, as the most prominent bacterial causes of bacteremia and sepsis, as well as *Candida albicans*, one of the most prominent causes of fungemia. Model systems including spiked negative blood samples, as well as positive blood cultures, without further culturing steps, were investigated. Furthermore, an experiment designed to determine the incubation time needed for correct identification of the infectious pathogens in blood cultures was performed. The results for the spiked negative blood samples showed that proteotyping was 100- to 1,000-fold more sensitive, in comparison with the MALDI-TOF MS-based approach. Furthermore, in the analyses of ten positive blood cultures each of *E. coli* and *S. aureus*, both the MALDI-TOF MS-based and proteotyping approaches were successful in the identification of *E. coli*, although only proteotyping could identify *S. aureus* correctly in all samples. Compared with the MALDI-TOF MS-based approaches, shotgun proteotyping demonstrated higher sensitivity and accuracy, and required significantly shorter incubation time before detection and identification of the correct pathogen could be accomplished.

## Introduction

Blood stream infections (BSIs) are ranked as the third leading cause of health care-related infections (Seymour et al., 2016). BSIs are caused mainly by bacteria or fungi and are frequently derived from urinary tract or abdominal infections or community acquired pneumonia (Angus et al., 2001; Lagu et al., 2012). Common causative bacterial agents of BSIs include *Escherichia coli*, *Staphylococcus* spp., *Enterococcus* spp., *Streptococcus* spp., *Pseudomonas aeruginosa*, and *Klebsiella* spp. (Martinez and Wolk, 2016). The presence of *Candida* fungi in blood, referred to as, “candidemia”, is also common in hospitalized patients (Alam et al., 2014; Klingspor et al., 2018). Most cases of candidemia are caused by five species: *Candida albicans*; *Candida glabrata*; *Candida parapsilosis*; *Candida tropicalis*; and *Candida krusei* (Pappas et al., 2003; Bassetti et al., 2013; Lindberg et al., 2019; Xiao et al., 2019). Among them, *C. albicans* is the most common fungus isolated from BSI in adults and children and is associated with high rates of mortality (Alam et al., 2014; Steinbach, 2016). Early identification of infectious strains and treatment with appropriate anti-microbial drugs are the keys to reducing morbidity and mortality associated with BSI (Metzgar et al., 2016; Tassinari et al., 2018), as BSI can lead to sepsis (Huerta and Rice, 2018), a serious and life-threatening condition of multiorgan failure, triggered by an uncontrolled host response to an infection (Singer et al., 2016). On a global scale, sepsis is one of the most predominant causes of death in hospitalized patients (Fleischmann et al., 2016a; Fleischmann et al., 2016b; Grumaz et al., 2016; Ibrahim et al., 2020), highlighting the importance of rapid diagnostics of BSIs.

The blood culture is still the “gold standard” for the diagnosis of patients with BSI (Mancini et al., 2010; Źródłowski et al., 2018; Ombelet et al., 2019). The identification of pathogenic microorganisms and antimicrobial susceptibility testing also generally relies on the cultivation and identification of pathogens from blood culture flasks (Opota et al., 2015). Positive blood cultures indicate a microbial growth (bacteria and/or fungi), whereas negative blood cultures indicate no microbial growth in the blood culture flasks. Completed routine identification may be achieved within two days but may take longer for infections by different species and different strains (Seng et al., 2009; Kirn and Weinstein, 2013; Nagy et al., 2018). Drawbacks exist in performing the cultivation step, including the time required for the culturing itself, as well as the fact that many blood cultures are inconclusive, in the sense that the bacteria or fungi from patient samples may not grow in the culture or cannot be recovered, *i.e.* false negative results (Murray and Masur, 2012; Skvarc et al., 2013; Sinha et al., 2018; Hazwani et al., 2020; Źródłowski et al., 2020). However, false negative blood cultures may result from the presence of antibiotics in the culture, originating from the patient blood, from infections caused by opportunistic microorganisms that grow poorly in standardized, automated, blood culture systems or that only few viable cells of the pathogen have been recovered from patient blood samples (Sinha et al., 2018). Furthermore, the success of recovery of microorganisms in cases of bacteremia has been shown to be linked to the volume of blood initially taken (Murray and Masur, 2012; Skvarc et al., 2013; Loonen et al., 2014; Opota et al., 2015; Henning et al., 2019). In some cases, however, it is not possible to recover large volumes of blood (Kirn and Weinstein, 2013), for example, from newborn infants at neonatal intensive care units, wherein culture-independent diagnostics methods, i.e., not relying on blood cultures, and thus not needing large volumes of patient blood, would be of utmost importance (Steinbach, 2016; Henning et al., 2019).

Recently, methods for detection of genetic material (Mancini et al., 2010; Skvarc et al., 2013; Gosiewski et al., 2014; Liesenfeld et al., 2014; van de Groep et al., 2018), as well as DNA-sequencing-based methods (Grumaz et al., 2016; Gosiewski et al., 2017; Watanabe et al., 2018; Grumaz et al., 2020), have been used for the detection of pathogens in blood. Serological methods, including detection of lipopolysaccharides for Gram-negative bacteria or galactomannan for fungi (Opal, 2010; Dickson and Lehmann, 2019), but also methods based on Gram-staining and fluorescence in-situ hybridization (FISH) have been successfully applied for direct detection of pathogens in blood (Gosiewski et al., 2014; Źródłowski et al., 2020). An advantage of these methods is that they do not rely on isolates from blood cultures and can be used on samples where antibiotic treatment has been initiated (Źródłowski et al., 2018; Źródłowski et al., 2020). These procedures are efficient, although some of them are relatively expensive and not suitable for the routine in clinical laboratories with large numbers of samples (Mancini et al., 2010; Skvarc et al., 2013; Opota et al., 2015; Źródłowski et al., 2018; Briggs et al., 2021).

Matrix assisted laser desorption/ionization time of flight mass spectrometry (MALDI-TOF MS)-based identification of microorganisms has emerged as an alternative or a complement to the traditional phenotypic methods (Seng et al., 2009; Ferroni et al., 2010; Welker and Moore, 2011; Spanu et al., 2012; Kondori et al., 2015). The implementation of MALDI-TOF MS identification into the clinical routine laboratories has been successful due to several benefits, including ease-of-use, speed in obtaining results, low cost, as well as high resolution of species identifications, in most cases (Florio et al., 2018). However, generally, the approach includes a necessary cultivation step, although efforts are being made to implement short cultivations steps or perform direct MALDI-TOF MS-based identification from the positive blood cultures (Florio et al., 2018; Briggs et al., 2021). A direct analysis of a patient sample, however, relies on successful removal of human blood cells and plasma proteins, as these may hinder the identification of bacterial and fungal pathogens, which are present to a much lesser degree in a blood sample, compared with the cells and proteins of human origin.

Even though the “gold standard” of blood cultures is nowadays complemented by molecular methods and MALDI-TOF MS approaches, none of the current rapid diagnostic methodologies is able to provide broad-range species identification as well as results regarding antibiotic susceptibility in one single analysis (Briggs et al., 2021). Therefore, development of reliable and rapid analytical techniques for comprehensive diagnostics and characterizations of infectious bacteria is still essential.

In this study, we investigate the use of unique peptides and bottom-up proteomics for performing rapid diagnostics of infectious bacteria and fungi. “Bottom-up proteomics”, as differentiated from “top-down proteomics”, relies on digestion of proteins into peptides using proteolytic enzymes, such as trypsin, followed by separation of the complex mixture of peptides, using a separation step, typically liquid chromatography (LC) prior to ionization, fragmentation and identification of peptides by tandem mass spectrometry (MS/MS) (Karlsson et al., 2015). Bottom-up proteomic approaches have been employed to increase the discriminative power and resolution of closely related species, i.e., to strain-level typing (Dworzanski et al., 2006; Karlsson et al., 2012; Chenau et al., 2014; Semanjski and Macek, 2016; Chen et al., 2019; Karlsson et al., 2020). Such “proteotyping” approaches, using peptide biomarkers, enable differentiating, for instance, the taxonomically close species of *Streptococcus pneumoniae*, *Streptococcus pseudopneumoniae* and *Streptococcus mitis* of the Mitis group of the genus *Streptococcus* (Karlsson et al., 2018). To facilitate the identification of species-unique peptides, several different bioinformatics pipelines have been developed to highlight peptides unique for different taxonomic levels (Family, Genus, Species) (Boulund et al., 2017; Grenga et al., 2019; Pible et al., 2020), including the Microorganism Classification and Identification (MiCId), which was used in this study (Alves et al., 2018; Alves and Yu, 2020). The goal of this study was to detect and identify bacteria and fungi directly in a model system including spiked negative blood samples, as well as in positive blood cultures, without further cultivation, using liquid chromatography tandem mass spectrometry (LC-MS/MS) and species-unique peptide identification, *i.e.* shotgun proteotyping (Karlsson et al., 2015; Karlsson et al., 2017; Karlsson et al., 2018; Karlsson et al., 2020).

## Material and Methods

### Experimental Design

**Figure 1:**
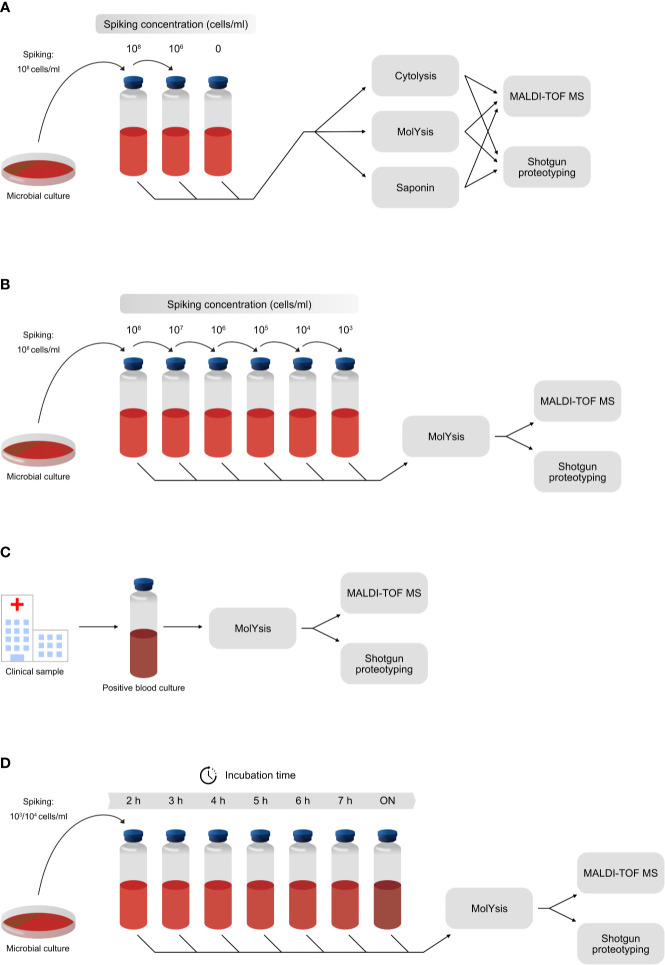
The experimental design of the experiments included in the study. **(A)** Host depletion methods MolYsis, Saponin and Cytolysis were investigated by spiking negative blood with different numbers of cells from *S. aureus*. **(B)** Assessment of the sensitivity of shotgun proteotyping, compared with direct MALDI-TOF MS-based identification. Different numbers of cells were added to negative blood samples in the range of 1,000 cells/ml to 100 million cells/ml, and the samples were then analyzed by both methodologies. **(C)** Assessment of the accuracy of shotgun proteotyping and direct MALDI-TOF MS-based identification by analysis of positive blood cultures from the clinical routine laboratories. **(D)** Assessment of the incubation time needed for a positive identification of the correct species, using shotgun proteotyping and direct MALDI-TOF MS-based identification. A low number of cells (1,000 or 10,000) was added to negative blood samples, followed by incubation in a blood culture cabinet. Samples were taken after 2, 3, 4, 5, 6 and 7 hours of incubation, as well as after overnight (ON).

Four different experiments were included, briefly outlined in **Figure 1**. The first experiment was designed to investigate a proper workflow for reducing cells and proteins of host origin, and therefore various host depletion methods were tested (**Figure 1A**). The next experiment was focused on assessing the sensitivity of the shotgun proteotyping approach, as compared to the traditionally used MALDI-TOF MS-based identification. This was performed by adding known amounts of bacterial or fungal cells to negative blood samples (**Figure 1B**). In the third experiment, positive blood cultures derived from patient samples, were analyzed to assess the accuracy of the shotgun proteotyping approach (**Figure 1C**). Finally, in order to investigate the time needed for correct identification of the infectious pathogens in blood cultures, a low number of bacterial or fungal cells (1,000 or 10,000) was added to blood culture flasks followed by incubation in a blood culture cabinet, and at each hour, from 2 to 7 hours, plus overnight (ON), samples were taken for analysis (**Figure 1D**). An overnight (ON) incubation corresponds to an hour range of 15-18 hours.

### Cultivation of Bacteria and Fungi

Bacterial and fungal strains were acquired from the Culture Collection University of Gothenburg (CCUG, www.ccug.se). *Staphylococcus aureus* and *Escherichia coli* were included as representative bacterial pathogens and *Candida albicans* was included as a representative fungal pathogen. *S. aureus* (CCUG 41582), *E. coli* (CCUG 49263) and *C. albicans* (CCUG 32723) were cultivated on Columbia agar supplemented with 5% of defibrinated horse blood (Substrate Unit, Department of Clinical Microbiology, Sahlgrenska University Hospital, Gothenburg, Sweden) and incubated at 37°C, for 24 hours.

### Host Biomass Depletion Methods: Saponin, MolYsis, and Cytolysis

Three different methods for host biomass depletion of the samples were investigated: A cytolysis approach, a protocol using Saponin and the use of a commercial kit (MolYsis Kit™ Basic5, Molzym, GmbH & Co. KG, Bremen, Germany) with a modified protocol. The Cytolysis approach relies on osmotic shock, lysis of human cells, but maintaining the integrity of bacterial and fungal cells, which are more resistant. The Saponin approach, which is used at the Department of Clinical Microbiology (Sahlgrenska University Hospital, Gothenburg, Sweden) for performing direct MALDI-TOF-MS-based analyses of blood samples, consists of adding a solution of saponin for lysis of host cells. The MolYsis kit has previously been used for identification of *S. aureus* in positive blood cultures (McCann and Jordan, 2014; Thoendel et al., 2016), for application of PCR-based methods. However, in the procedure, the first step of the MolYsis workflow entails selective lysis of human blood cells, whereby a pellet of bacteria or fungi is achieved. The later steps of the procedure involve lysis of the cells and extraction of DNA, although, in this study, the MolYsis procedure was used only for removal of human cells and the generation of a pellet of bacteria or fungi, as described previously (Karlsson et al., 2020). The pellet of bacteria or fungi was then processed, using a separately developed protocol, as described.

### MolYsis Clean-Up

The MolYsis™ Kit (Molzym, GmbH & Co. KG, Germany) was used, according to the manufacturer´s instructions, with some adjustments (Karlsson et al., 2020). After centrifugation at 12,000 x g for 5 minutes, the supernatant was discarded, and the pellet saved. MALDI-TOF MS was performed on the bacterial pellets. In cases where pellets were not large enough to be processed, they were dissolved in 5 µl of deionized water (W6-212 Water, Optima^®^ LC/MS, Fisher Chemical), whereas the pellet for proteotyping was dissolved in 150 µl of PBS.

### Saponin Clean-Up

An in-house Saponin clean-up method, modified from a protocol (Ferroni et al., 2010) was implemented. Saponin solution (200 μl of 5% solution in distilled water) was added to 1 ml of blood culture sample. The suspension was vortexed and then allowed to stand at room temperature for 5 minutes. The suspension was then centrifuged at 12,000 x g for 1 minute. The pellet was washed 3 times by resuspension with 1 ml distilled water and centrifugation at 12,000 x g for 5 minutes. The final supernatant was discarded, and the pellet was saved. MALDI-TOF MS was performed on the bacterial pellets. In cases where pellets were not large enough to be processed, they were dissolved in 5 µl of deionized water (W6-212 Water, Optima^®^ LC/MS), while the pellet for proteotyping was dissolved in 150 µl of PBS.

### Cytolysis Clean-Up

Cell lysis by osmotic shock (Cytolysis) was performed. The blood culture sample was centrifuged at 12,000 x g for 5 minutes. The supernatant was discarded, and 1 ml deionized water was added to resuspend the pellet, to create osmotic shock for the blood cells, lysing them, but leaving bacterial and fungal cells intact. Samples were centrifuged at 12,000 x g for 1 minute. The supernatant was discarded, and the pellet saved. MALDI-TOF MS was performed on the bacterial pellets. In cases where pellets were not large enough to be processed, they were dissolved in 5 µl of deionized water (W6-212 Water, Optima^®^ LC/MS), while the pellet for proteotyping was dissolved in 150 µl of PBS.

### The Sensitivities and Specificities of MALDI-TOF-MS and Shotgun Prototyping

Horse blood was spiked with bacterial or fungal cells in 10-fold dilutions, generating a range of cell concentrations from 0 to 100 million cells/ml, to assess the sensitivity of the MALDI-TOF MS and nanoLC-MS/MS shotgun proteotyping methods. The final concentrations of bacteria or fungi in blood ranged from 0 up to 10^8^ cells/ml (0, 10, 100, 1,000, 10,000, 100,000, 1 million, 10 million, 100 million cells/ml).

### Preparation of Samples Spiked With Bacterial or Fungal Cells

Bacterial and fungal biomass were collected from agar plates and resuspended in phosphate-buffered saline (PBS). Bacterial and fungal cell densities (Optical Density, OD) were measured by spectrophotometry (WPA CO 8000 Cell Density Meter, Biochrom Ltd. Cambridge, United Kingdom) at a wavelength of 600 nm. For each experiment, the same amounts of biomass were established, by adjusting the OD to 1.0 (OD = 1.0 corresponds to ~10^8^ bacteria) in 1 ml of PBS. The biomass was washed with PBS three times by centrifuging the sample for 5 minutes at 12,000 x g, discarding the supernatant and resuspending the pellet in 1 ml of PBS. Finally, the biomass was centrifuged for 5 minutes at 12,000 x g and supernatant discarded. The pellet was resuspended in 100 µl PBS, which was added to 900 µl sterile horse blood, creating the first spiked sample of 1 ml blood containing 10^8^ bacterial cells per ml. This procedure was followed to create samples containing bacterial/fungal cells with 10-fold serial dilutions of 0 cells/ml to 100 million cells/ml (10^8^). The spiking procedure was performed by generating two samples in parallel. In the final step, the two samples were combined, vortexed and split, in order to ensure that the contents of each portion were as equivalent as possible, i.e., to ensure the same number of cells in each sample. One of the samples was used for MALDI-TOF MS analysis and the other for proteotyping by tandem nanoLC-MS/MS.

### Incubation Time Required for Direct Identification of Bacteria and Fungi by MALDI-TOF MS or Shotgun Proteotyping

The kinetics of direct identifications of bacteria and fungi by MALDI-TOFMS or proteotyping were studied by incubation of bacteria and fungi at a final concentration of 1,000 cells/ml (or 10,000 cells/ml) in the blood culture flasks (BACT/ALERT^®^FA Plus Aerobic 30 ml, bioMérieux, Marcy l’Etoile, France). The blood cultures were incubated in a continuous monitoring blood culture system (CMBCS) with a colorimetric sensor (BacT/Alert^®^; bioMérieux, Marcy l’Etoile, France) (Kennedy et al., 1995). The samples were collected from the blood culture tubes at different time points after incubation [2 h, 3 h, 4 h, 5 h, 6 h, 7 h and overnight (ON)]. Prior to analysis, the samples were subjected to the MolYsis clean-up procedure. The samples for MALDI-TOF MS were stored at 2°C until analysis or, in the case of proteotyping, were stored at -20°C until analysis by tandem nanoLC-MS/MS for detection of microbial pathogen in blood samples.

### Analysis of Positive Blood Cultures

Positive blood cultures with *E. coli* (n=10), *S. aureus* (n=10) and *C. albicans* (n=5) were included in this study. The samples were collected at the Department of Clinical Microbiology, Sahlgrenska University Hospital in Gothenburg, Sweden. Only samples that were collected as part of the standard diagnostic protocols were included in this study; no additional or extra sampling from patients was carried out and no patient identifiable information was collected; hence, informed consent was not required. Blood cultures were incubated by means of a BacT/Alert continuous monitoring blood culture system (CMBCS) that detects bacterial or fungal growth. Bottles flagged as positive by the BacT/Alert system were sub-cultured and incubated at 37°C, under aerobic and anaerobic conditions, until positive growth or otherwise, for 7-10 days, and interpreted according to the standard protocols in the laboratory. Prior to proteotyping MS analysis, the samples were subjected to the MolYsis clean-up procedure.

### Matrix Assisted Laser Desorption/Ionization Time of Flight Mass Spectrometry (MALDI-TOF MS) Analysis

MALDI-TOF MS was performed on the bacterial pellets. In cases where pellets were not large enough to be processed, they were dissolved in 5 µl of deionized water (W6-212 Water, Optima^®^ LC/MS). Each sample was spotted in four replicates on disposable target slides (VITEK^®^ MS-DS slide, bioMérieux, France). After drying at room temperature, 1 µl of ready-to-use α-cyano-4-hydroxycinnamic acid (CHCA) matrix solution (VITEK^®^ MS-CHCA, bioMérieux, France) was added and allowed to dry at room temperature. In the cases of Gram-positive bacteria (*S. aureus*) and fungi samples (i.e., *C. albicans*), prior to the addition of the CHCA matrix solution, 1 µl of a formic acid solution (70%, VITEK^®^ MS-FA, bioMérieux, France) was added to the sample and allowed to dry at room temperature. Subsequently, the slides were placed in the MALDI-TOF MS (VITEK MS™ RUO v.3.0, bioMérieux, France) with standard settings for routine identification, in a mass range of 2 to 20 kDa, Research Use Only (RUO), to be analyzed. Controls in the slide were performed in each run with *E. coli* CCUG 10979. The resulting spectra were analyzed in the proprietary IVD (*In Vitro* Diagnostics) Knowledge Database v2 (bioMérieux, France).

### Sample Preparation for Proteotyping

After the host biomass depletion methods (Saponin, Cytolysis or MolYsis), the pellets containing bacterial or fungal cells were resuspended in 150 µl of PBS. The cell suspensions were transferred to 200 µl vials containing glass beads (Sigma-Aldrich, G1145). The cells were lysed by bead-beating, using approx. 50 μl of acid-washed 150-212 μm glass beads in a 200 μl tube, with a TissueLyser (Qiagen, 85220) with the following settings: frequency 1/25 s and 5 minutes. The cell lysates were frozen at -20°C until analysis.

### Digestion of Samples for Proteotyping Into Peptides

Samples were thawed and sodium deoxycholate (SDC, 5%) was added to a 1% final concentration. Trypsin (2 µg/ml, 100 µl ammonium bicarbonate, 20 mM pH 8) was added and samples were digested overnight (ON) at 37°C. SDC was removed by acidification with formic acid (neat; 2 µl to 100 µl sample) and the supernatant was stored in -20°C until analysis.

### nanoLC-MS/MS Analysis of Proteotyping Samples

Peptide samples were desalted, using PepClean C18 spin columns (Thermo Fisher Scientific), according to the manufacturer’s guidelines, prior to analysis on a Q Exactive HF mass spectrometer (Thermo Fisher Scientific) interfaced with Easy nLC 1200 liquid chromatography system (Thermo Fisher Scientific). Peptides were trapped on an Acclaim Pepmap 100 C18 trap column (100 μm x 2 cm, particle size 5 μm, Thermo Fischer Scientific) and separated on an in-house packed analytical column (75 μm x 300 mm, particle size 3 μm, Reprosil-Pur C18, Dr. Maisch), using a linear gradient from 7% to 35% B over 45 or 75 minutes, followed by an increase to 100% B for 5 minutes at a flow of 300 nL/minutes, where solvent A was 0.2% formic acid in water and solvent B was 0.2% formic acid, 80% acetonitrile in water. MS/MS analysis was performed in a data-dependent mode where the precursor ion mass spectra were acquired at a resolution of 60,000, *m/z* 400-1,600, and the Top10 most intense precursor ions, with charge states of 2 to 4, were selected for fragmentation. The isolation window was set to 1.2 Da, and MS2 spectra were recorded at a resolution of 30,000, *m/z* 200-2,000. Dynamic exclusion was set to 20 seconds and 10 ppm.

### MiCId Bioinformatics Pipeline for Microorganism Classification and Identification

Microorganism Classification and Identification (MiCId) is a workflow designed for the identifications of microorganisms, proteins and estimations of microbial biomass in samples (Alves et al., 2016; Alves et al., 2018; Alves and Yu, 2020). For a rapid identification of microorganism, MiCId (version v.06.11.2020) workflow performs peptide identification by querying the MS/MS spectra in a peptide-centric database and assigns to every peptide a MS/MS spectrum-specific measure, namely, E- value (Alves et al., 2007; Alves et al., 2008; Alves and Yu, 2008; Alves et al., 2010). To provide microorganism identification significances, MiCId computes a weighted unified E-value by combining the spectrum-specific E-values of the identified peptides mappable to a given microorganism. For each identified microorganism, MiCId also computes a prior probability using a modified expectation-maximization method. The computed prior probabilities reflect the relative protein biomasses, due to the various reported microorganisms, in the sample. Assigning to microorganism accurate E-values along with the prior probability, MiCId provides users a measure suitable for controlling false positives (type I errors). In MiCId’s default settings, microorganisms identified with E-values smaller or equal to 0.01 and with prior probability greater or equal to 0.01 are deemed true positives. Using these cut-off values allows users to control the false positive rate well below 5%.

Since peptides that are unique to a taxon at a given taxonomic level are often used as the main evidence for the presence of that taxon, a false identification of such unique peptides can have undesirable consequence. To better control false microorganism identification, in addition to computing a unified E-value mentioned above, MiCId put an extra requirement for an identified peptide to qualify as an unique peptide. An identified unique peptide to a given taxon must have an E-value 10^-4^ or less aside from uniquely mappable to that taxon (Alves et al., 2018).

The database MiCId used to query MS/MS spectra comprises 3,887 organisms, including *Homo sapiens* and *Equus caballus*, covering 1,959 species. Protein sequences included in the database, for the 3,868 organisms (excluding *Shigella*), were downloaded (on April 27, 2020) from the National Center for Biotechnology Information (NCBI) at (ftp://ftp.ncbi.nlm.nih.gov/genomes/genbank/).

**Supplementary Table 7S** lists the microorganisms included. When performing database searches, up to two missed cleavage sites per peptide were allowed under the digestion rules of trypsin and Lys-C. The amino acid cysteine was kept unmodified. The mass error tolerance of 5 ppm was set for precursor ions and 20 ppm for product ions.

## Data Availability

The mass spectrometry proteomics data have been deposited to the ProteomeXchange Consortium *via* the PRIDE (Perez-Riverol et al., 2019) partner repository with the dataset identifier, PXD023033.

## Results

### Host Biomass Depletion Methods of Blood Samples

Three different host biomass depletion methods were employed on blood samples spiked with *S. aureus* (CCUG 41582), wherein the MolYsis™ kit was observed to reduce the number of peptides from horse blood origin (n= 17, 44 and 62 at 0, 10^6^ and 10^8^ cells respectively) while also recovering bacterial peptides (n=0, n=6 and n=415 from 0, 10^6^ and 10^8^ cells, respectively) (**Table 1**). The computed prior probabilities reflect the relative protein, due to the various reported (micro)organisms in the sample and can be used to assess the performance of the clean-up protocols. **Table 1** shows that, for the horse blood spiked samples with 10^6^ and 10^8^ cells/ml, the relative sample biomass for *S. aureus* are respectively around 25% and 93% *via* MolYsis, around 5% and 75% *via* Saponin, and around 0% and 60% *via* Cytolysis (**Table 1** and **Supplementary Table 1S**). MALDI-TOF MS (results not shown in the table) only identified *S. aureus* when 10^8^ cells/ml were added to the blood samples.

**Table 1:** Assessment of host biomass depletion methods prior to MS analyses.

Spiking concentration (cells/ml)	Host biomass depletion method	Taxa identified	Identification fraction	Average ln(E-value)	Average number of identified unique peptides	Average number of identified peptides	Average Prior
0	MolYsis kit	*H. sapiens*	1/1	-223	43	154	0.693
		*E. caballus*	1/1	-62.2	17	104	0.282
		*S. aureus*	0/1	–	–	–	–
	Saponin	*H. sapiens*	0/1	–	–	–	–
		*E. caballus*	1/1	-2440	663	1326	0.993
		*S. aureus*	0/1	–	–	–	–
	Cytolysis	*H. sapiens*	0/1	–	–	–	–
		*E. caballus*	1/1	-2780	786	1534	0.99
		*S. aureus*	0/1	–	–	–	–
10^6^	MolYsis kit	*H. sapiens*	3/3	-214	41 ± 14	173 ± 34	0.351 ± 0.02
		*E. caballus*	3/3	-175	44 ± 4	172 ± 23	0.366 ± 0.07
		*S. aureus*	3/3	-122	6 ± 1.4	117 ± 39	0.25 ± 0.05
	Saponin	*H. sapiens*	0/1	–	–	–	–
		*E. caballus*	1/1	-2790	756	1722	0.937
		*S. aureus*	1/1	-146	10	134	0.0544
	Cytolysis	*H. sapiens*	0/1	–	–	–	–
		*E. caballus*	1/1	-2500	704	1295	0.989
		*S. aureus*	0/1	–	–	–	–
10^8^	MolYsis kit	*H. sapiens*	3/3	-113	23 ± 5	118 ± 19	0.0199 ± 0.003
		*E. caballus*	3/3	-302	62 ± 13	187 ± 28	0.0521 ± 0.003
		*S. aureus*	3/3	-5090	415 ± 78	3394 ± 681	0.926 ± 0.005
	Saponin	*H. sapiens*	0/1	–	–	–	–
		*E. caballus*	1/1	-1800	443	1015	0.248
		*S. aureus*	1/1	-5140	426	3417	0.751
	Cytolysis	*H. sapiens*	0/1	–	–	–	–
		*E. caballus*	1/1	-2700	651	1300	0.399
		*S. aureus*	1/1	-3820	265	2271	0.599

MiCId identification results of horse blood samples spiked with 0, 10^6^ and 10^8^ cells of S. aureus and processed with three different host biomass depletion methods.

MALDI-TOF MS (results not shown in the table) identified S. aureus only when 10^8^ cells/ml were added to the blood samples. In that case, the identification was successful with all three host biomass depletion methods. The denominator of the identification fraction shows the number of analyzed samples and the numerator shows the number of times each taxon has been identified; the number after “±” is the standard deviation (from triplicate analyses). For a taxon reported by MiCId, the “average number of identified peptides” records the average total number of identified peptides mappable to that taxon, while the “average number of identified unique peptides” sums to the total number of identified peptides satisfying the following two conditions simultaneously: these peptides must be unique to that taxon at a given taxonomic level and must have E-values that are 10^-4^ or less.

### The Sensitivities and Specificities of MALDI-TOF MS and Shotgun Proteotyping

The sensitivities and specificities of the proteotyping approach were investigated by adding (spiking) known amounts of bacterial or fungal cells, ranging from 0 to 100 million cells/ml to negative horse blood samples. The MALDI-TOF MS approach correctly identified the bacteria in the spiked samples containing the highest amounts of cells, i.e., 100 million cells/ml, but was not able to detect bacteria at 10 million cells/ml or less. In contrast, proteotyping was able to detect and identify species-unique peptides of the studied pathogens (*E. coli*, *S. aureus* and *C. albicans*) even down to spiked samples with as low as 10,000 cells/ml. Thus, proteotyping, was a 100- to 1,000-fold more sensitive in comparison of MALDI-TOF MS (**Table 2** and **Supplementary Table 2S**).

**Table 2:** The sensitivity of the LC-MS/MS shotgun proteotyping protocol.

Spiked species	Taxa identified	Spiking concentration (cells/ml)			
		10^4^	10^5^	10^6^	10^7^
*S. aureus*	Species (*S. aureus*)	0/3	0/3	3/3	
	Genus (*Staphylococus*)	0/3	1/3	3/3	
	Family (*Staphylococcaceae*)	0/3	1/3	3/3	
	Order (Bacillales)	0/3	1/3	3/3	
	Class (Bacilli)	0/3	1/3	3/3	
	Phylum (Firmicutes)	0/3	2/3	3/3	
*E. coli*	Species (*E. coli*)	3/3	3/3	3/3	
	Genus (*Escherichia*)	3/3	3/3	3/3	
	Family (*Enterobacteriaceae*)	3/3	3/3	3/3	
	Order (Enterobacterales)	3/3	3/3	3/3	
	Class (Gammaproteobacteria)	3/3	3/3	3/3	
	Phylum (Proteobacteria)	3/3	3/3	3/3	
*C. albicans*	Species (*C. albicans*)	0/3	3/3	3/3	3/3
	Genus (*Candida*)	0/3	3/3	3/3	3/3
	Family (*Saccharomycetaceae*)	0/3	3/3	3/3	3/3
	Order (Saccharomycetales)	0/3	3/3	3/3	3/3
	Class (Saccharomycetes)	0/3	3/3	3/3	3/3
	Phylum (Ascomycota)	0/3	3/3	3/3	3/3

MiCId identification results of S. aureus, E. coli and C. albicans spiked at different concentrations in horse blood.

The number x/y in the table is the identification fraction, in which the denominator shows the number of samples containing the microorganism and the numerator is the number of times that microorganism is identified correctly. E. coli and S. aureus were analyzed through the concentration ranges of 10^3^-10^6^ cells/ml, whereas C. albicans was analyzed through concentration ranges of 10^3^-10^7^ cells/ml. MALDI-TOF MS (results not shown in the table) identified S. aureus, E. coli and C. albicans only when 10^8^ cells/ml were added to the blood samples.

### Analysis of Positive Blood Cultures

Samples from ten positive blood cultures from *E. coli* and *S. aureus* and five positive blood cultures from *C. albicans* were analyzed directly, without further culturing, using MALDI-TOF MS-based analysis and proteotyping. **Table 3** shows the identifications from the direct MALDI-TOF MS analyses, as well as the number of species-unique peptides found by the proteotyping analysis. Both protocols were able to identify the correct species in all ten samples with *E. coli*. However, MALDI-TOF MS was able to identify *S. aureus* in only four of the ten samples, whereas proteotyping was able to correctly identify *S. aureus* in all ten samples. *C. albicans* was not identified using the direct MALDI-TOF MS-based approach, however, proteotyping successfully identified the correct species in 4 out of the 5 samples (**Table 3**).

**Table 3:** Comparison of identification accuracies of MALDI-TOF MS and shotgun proteotyping.

Species identified in the routine clinical laboratories	Sample ID	Shotgun proteotyping ID (MiCId)				Direct MALDI-TOF MS ID
		Species	Ln(E-value)	Number of identified unique peptides	Prior	
*E. coli*	E1	*E. coli*	-1.413e+02	3	6.356e-01	*E. coli*
	E2	*E. coli*	-3.877e+02	14	7.162e-01	*E. coli*
	E3	*E. coli*	-1.605e+02	3	3.976e-01	*E. coli*
	E4	*E. coli*	-3.024e+03	96	5.957e-01	*E. coli*
	E5	*E. coli*	-1.118e+02	2	3.694e-01	*E. coli*
	E6	*E. coli*	-2.224e+02	6	7.232e-01	*E. coli*
	E7	*E. coli*	-2.181e+03	68	9.299e-01	*E. coli*
	E8	*E. coli*	-4.306e+02	14	7.500e-01	*E. coli*
	E9	*E. coli*	-9.672e+02	30	7.335e-01	*E. coli*
	E10	*E. coli*	-7.703e+02	23	6.926e-01	*E. coli*
*S. aureus*	S1	*S. aureus*	-8.468e+01	11	4.528e-02	Negative
	S2	*S. aureus*	-3.991e+02	38	9.623e-02	Negative
	S3	*S. aureus*	-1.368e+03	113	3.798e-01	Negative
	S4	*S. aureus*	-2.098e+03	151	3.965e-01	*S. aureus*
	S5	*S. aureus*	-4.020e+03	303	6.842e-01	*S. aureus*
	S6	*S. aureus*	-8.035e+02	67	1.903e-01	Negative
	S7	*S. aureus*	-2.856e+02	20	9.698e-02	Negative
	S8	*S. aureus*	-8.655e+02	75	2.279e-01	*S. aureus*
	S9	*S. aureus*	-1.824e+02	25	6.388e-02	Negative
	S10	*S. aureus*	-3.495e+03	261	6.393e-01	*S. aureus*
*C. albicans*	C1	*C. albicans*	-9.763e+01	14	1.150e-01	Negative
	C2	*C. albicans*	-2.334e+02	26	3.706e-01	Negative
	C3	*C. albicans*	-1.896e+01	2	1.696e-02	Negative
	C4	*C. albicans*	-9.807e+01	10	6,586e-01	Negative
	C5	Negative	–	–	–	Negative

Results of analysis of ten positive blood cultures for E. coli and S. aureus and five for C. albicans, using MALDI-TOF MS and proteotyping. The identifications by MALDI-TOF MS, as well as the number of species-unique peptides are shown.

### Incubation Time Required for Direct Identification of Bacteria and Fungi by MALDI-TOF MS or Proteotyping

Direct MALDI-TOF MS detected and identified *E. coli* in bacterial positive blood culture flasks after incubation overnight, however, *S. aureus* and *C. albicans* were not detected by MALDI-TOF MS in any of the blood cultures at any time after incubation (**Table 4**). Proteotyping was able to detect *S. aureus* after 7 hours of incubation, and for *E. coli* a correct identification could be achieved after only 5 hours of incubation. *C. albicans* was correctly identified by proteotyping after ON incubation (**Table 4** and **Supplementary Table 4S**).

**Table 4:** Incubation times for identifications by shotgun proteotyping.

Spiked species	Spiking concentration (cells/ml)	Taxa identified	Incubation time in blood culture cabinet						ON
			2 h	3 h	4 h	5 h	6 h	7 h	
*E. coli*	1,000	*E. coli*	0/2	0/2	0/2	**1/2**	**2/2**	**2/2**	**2/2**
		*Escherichia*	0/2	0/2	0/2	**1/2**	**2/2**	**2/2**	**2/2**
		*Enterobacteriaceae*	0/2	0/2	0/2	**1/2**	**2/2**	**2/2**	**2/2**
		Enterobacterales	0/2	0/2	0/2	**1/2**	**2/2**	**2/2**	**2/2**
		Gammaproteobacteria	0/2	0/2	0/2	**1/2**	**2/2**	**2/2**	**2/2**
		Proteobacteria	0/2	0/2	**1/2**	**1/2**	**2/2**	**2/2**	**2/2**
*S. aureus*	1,000	*S. aureus*	0/2	0/2	0/2	0/2	0/2	**1/2**	**2/2**
		*Staphylococus*	0/2	0/2	0/2	0/2	0/2	**1/2**	**2/2**
		*Staphylococcaceae*	0/2	0/2	0/2	0/2	0/2	**1/2**	**2/2**
		Bacillales	0/2	0/2	0/2	0/2	0/2	**1/2**	**2/2**
		Bacilli	0/2	0/2	0/2	0/2	0/2	**1/2**	**2/2**
		Firmicutes	0/2	**1/2**	0/2	0/2	0/2	**1/2**	**2/2**
*S. aureus*	10,000	*S. aureus*	0/2	0/2	0/2	**2/2**	**2/2**	**2/2**	**2/2**
		*Staphylococus*	0/2	0/2	0/2	**2/2**	**2/2**	**2/2**	**2/2**
		*Staphylococcaceae*	0/2	0/2	0/2	**2/2**	**2/2**	**2/2**	**2/2**
		Bacillales	0/2	0/2	0/2	**2/2**	**2/2**	**2/2**	**2/2**
		Bacilli	0/2	0/2	0/2	**2/2**	**2/2**	**2/2**	**2/2**
		Firmicutes	0/2	**1/2**	0/2	**2/2**	**2/2**	**2/2**	**2/2**
*C. albicans*	1,000	*C. albicans*	0/1	0/1	0/1	0/1	0/1	0/1	**2/2**
		*Candida*	0/1	0/1	0/1	0/1	0/1	0/1	**2/2**
		*Saccharomycetaceae*	0/1	0/1	0/1	0/1	0/1	0/1	**2/2**
		Saccharomycetales	0/1	0/1	0/1	0/1	0/1	0/1	**2/2**
		Saccharomycetes	0/1	0/1	0/1	0/1	0/1	0/1	**2/2**
		Ascomycota	0/1	0/1	0/1	0/1	0/1	0/1	**2/2**

Incubation time needed for accurate identifications of E. coli, S. aureus and C. albicans, using shotgun proteotyping after incubation of 1,000 (or 10,000) cells in negative blood samples and incubation in blood culture cabinets.

The number x/y in the table is the identification fraction, in which the denominator shows the number of samples containing the microorganism and the numerator is the number of times that microorganism is identified correctly at different taxonomic levels. In the analyses of S. aureus, a false positive identification at the Firmicutes level was observed in one of the duplicates at 3 h incubation.

Bold signifies a positive identification in at least one of the replicates.

## Discussion

Early recognition of BSIs is crucial for successful treatment of patients, before conditions worsen and, possibly, become fatal, by development of sepsis (Kumar et al., 2006; Loonen et al., 2014). Clinical manifestations of sepsis are variable, depending upon sites of infection and causative microorganisms, as well as underlying conditions of patients (Iskander et al., 2013; Huang et al., 2019; Özenci et al., 2019). Unfortunately, diagnosis of sepsis is complex and problematic, often delayed because early symptoms are not recognized; many symptoms are subtle and mimic other clinical conditions (Iskander et al., 2013). While sepsis may be identified by clinical signs and symptoms in a patient, no “gold standard” diagnostic test exists (Singer et al., 2016; Ibrahim et al., 2020).

Rapid diagnoses of bloodstream infections, helping physicians administer proper treatments, is essential for reducing mortality due to BSIs and sepsis, as well as reducing costs associated with hospitalized patients. Since bloodstream infections are most often caused by a single pathogenic species (monospecies infection), and only rarely caused by two or several pathogens (Bouza et al., 2013), efforts have been focused on being able to skip the isolation and sub-culturing steps and analyze the positive blood cultures directly (Ferroni et al., 2010; Stevenson et al., 2010; Radic et al., 2016; Salimnia et al., 2016; Florio et al., 2018; Briggs et al., 2021). However, the use of MALDI-TOF MS for identification of the pathogen requires in most cases, a pure culture isolate of the bacteria or fungi after observation of a positive blood culture in the cultivation step (Opota et al., 2015). Currently, blood cultures and PCR-based assays are the protocols used for detecting and identifying the agents responsible for bloodstream infections (Book et al., 2013). PCR-based gene-amplification methods are potentially faster, however, a disadvantage is that they require pre-defined targets, which is a not neglectable limitation since the range of unknown infectious agents is extensive. Furthermore, at the time of diagnosis, the responsible microorganisms may no longer be in the bloodstream or are otherwise not detectable with existing methods (Warhurst et al., 2015).

In this study, the concept of using shotgun proteotyping, i.e., bottom-up proteomics, and peptide biomarkers, for detection of bloodstream infectious pathogens, was investigated. The bioinformatics pipeline MiCId, was used for data evaluation and identification of taxonomically unique peptides (Alves et al., 2018). Shotgun proteotyping does not rely on the traditionally applied cultivation step to obtain a pure culture isolate, instead, the samples can be analyzed directly. A key step in the success of using mass spectrometry-based proteomics for discovery of pathogens directly in clinical samples, is the removal of human “contamination”, such as blood cells and plasma proteins. The presence of these highly abundant proteins may hinder the detection and identification of peptides originating from the pathogens, which are present in much lower abundance in the samples. Different host biomass depletion methods were applied, i.e., clean-up by osmotic shock (Cytolysis), Saponin-based cell lysis protocol (Ferroni et al., 2010) and a commercial kit (MolYsis kit Basic5). The MolYsis kit not only facilitated the discovery of a high number of the bacterial peptides after the clean-up, but greatly reduced the number of peptides originating from host blood, compared to the other two approaches (**Table 1**). This agrees with earlier studies, where the MolYsis kit was used for identification of *S. aureus* in positive blood cultures (McCann and Jordan, 2014; Thoendel et al., 2016); therefore, the MolYsis kit with a modified protocol (Karlsson et al., 2020) was used as a host biomass depletion method throughout the experiments included in this study.

When blood samples spiked with cells of bacteria and fungi were analyzed with both MALDI-TOF MS and with proteotyping, the MALDI-based approach was able to correctly identify the species when the highest numbers of cells were added to the blood sample (100 million cells), whereas the proteotyping approach was able to find species-unique peptides from as few as 10,000-100,000 cells, demonstrating a thousand-fold increase in the sensitivity. This was expected, since the MALDI-TOF MS identification needs a certain amount of biomass for the generation of good quality spectra, which can be matched against the spectral database. Analyses of positive blood cultures by proteotyping were able to correctly identify all the positive blood cultures, with high numbers of species-unique peptides from each sample. The MALDI-based approach was also able to identify all the positive blood cultures containing *E. coli*, but only 4 of 10 positive blood cultures were correctly identified for those containing *S. aureus*. This is in agreement with earlier studies, showing that the identification of Gram-positive bacteria, such as *S. aureus*, using direct MALDI-TOF MS-based identification (i.e., no isolation of bacteria by a sub-culture from the positive blood cultures) fails more often, thus producing false negatives, compared to the identifications of Gram-negative bacteria, including *E. coli* (Ferroni et al., 2010; Stevenson et al., 2010; Loonen et al., 2012; Kirn and Weinstein, 2013; Briggs et al., 2021). Interestingly, in general, a higher number of species-unique peptides was detected and identified for *S. aureus* compared to the *E. coli* in the ten positive blood cultures. The higher number of species-unique peptides most likely reflects the taxonomy of the two species, *S. aureus* being more separated from closely related species, and, thus, having a larger repertoire of species-unique peptides compared to *E. coli*, making it easier to identify a higher number of species-unique peptides (Boulund et al., 2017).

Proteotyping relies on the identification of unique peptides (at any taxonomic level) and thus rely on accurate and comprehensive, often manually curated databases. Falsely identified unique peptides thus have far-reaching adverse consequence. In this study, MiCId was used as a bioinformatics pipeline to minimize the need of human curation and intervention. As explained in the Materials and Methods section, MiCId demands a more stringent criterion (E-value < 10^-4^) for qualifying unique peptides. This effectively removes false positives in terms of microorganism identification when only unique peptides will be employed for taxon identification. By limiting the count of unique peptides to those of high identification confidence, one may expect that the sensitivity in microorganism identification will drop. In other words, the occurrence of false negatives. MiCId mitigates this issue by offering the unified microorganism E-value that combines all identified peptides mappable to that microorganism, not just the unique peptides. This strengthen the signal of a microorganism if it is present in the sample, hence reduces the false negatives while controlling the false positives.

The analysis of positive blood cultures suspected to contain *C. albicans* were negative when analyzed by the direct MALDI-TOF MS-based method. This could be because *C. albicans* may be at a different growth state in the blood cultures, compared to when grown on agar medium. Therefore, protein expression levels might differ and, hence, the spectra generated might not match the spectra in the databases. Furthermore, a higher background of blood proteins of host origin, is expected from samples drawn from blood culture flasks, as compared to cultures grown on an agar plate. As this study was not focused on improving the MALDI-based approach, but rather to demonstrate the ability of proteotyping to correctly identify not only bacteria, but also fungi, no further efforts were performed to improve the results from the MALDI-based approach. The identification of fungi by MALDI-TOF MS-based methods often benefits from expanded extraction protocols, however, this was not implemented in this study, as it was not part of the clinical laboratory routine. Proteotyping was able to correctly identify 4 of the 5 samples included in the study. Further work is therefore necessary to optimize the sample clean-up step, and hence improve the accuracy in the proteotyping workflow for detection and identification of *C. albicans* in blood.

A clear difference was seen in both sensitivity and identification accuracy when comparing direct MALDI-TOF MS with shotgun proteotyping. Since it was suspected that lower numbers of bacterial and fungal cells (biomass) were needed for being able to correctly identify the infectious pathogens, an experiment was performed, to investigate if it was possible to reduce the time needed for performing a correct identification, i.e., even before the blood culture cabinets gave off an alarm. Generally, bacterial and fungal growth in blood culture flasks was detected after overnight incubation in blood culture cabinet (BacT/Alert^®^). Here, we studied the limit of detection and identification of bacterial and fungal growth in blood culture flasks, by incubating 1,000 cells/blood flask for 2, 3, 4, 5, 6 and 7 h incubation, as well as overnight (ON). The MALDI-TOF MS-based method was able to correctly identify *E. coli* only after ON incubation and was not able to identify *S. aureus* and *C. albicans*, even after ON incubation. On the other hand, proteotyping was able to identify *E. coli* even after 4-5 h of incubation, *S. aureus* at 7 h incubation, and *C. albicans* at the ON incubation (**Table 4**). The early identification of *E. coli* (4-5 hours), compared with *S. aureus* and *C. albicans* could, in these experiments, be explained by the shorter doubling time of *E. coli*, however further studies are required to pinpoint the influence of the growth rates of different species on the time needed for identification by proteotyping. Furthermore, since highly abundant housekeeping proteins are taxonomically more conserved within Families/Genera they are easier to detect compared to identifying lower abundant unique peptides at the species level. Therefore, species-unique peptides in combination with peptides on higher taxonomic levels should be used in the diagnosis. These peptides are shared by different species e.g. within the same genus, but still they will provide important information and strengthen the identification of the correct species and improves the sensitivity of the analysis. For example, an earlier detection of higher taxonomic level peptide biomarkers, e.g., *Enterobacteraceae*, would be of great value in reducing precious time spent for reaching a diagnosis during a suspected sepsis. In this study, an ON incubation was used for the digestion of proteins into peptides, although recently, it has been shown that the digestion time required for processing samples in proteotyping workflows can be reduced to 15 minutes (Hayoun et al., 2019). The proteotypic workflow can also be optimized further by implementing a targeted LCMS approach of the proteotypic peptides using triple quadrupole MS instrumentation (already present in many clinical laboratories) eliminating the database matching step.

Typically, addressing bloodstream infection is done through treatment with broad-spectrum antibiotics (Kuti et al., 2008; Tassinari et al., 2018). The global range of bacteria resistant to multiple antibiotics, particularly pathogens of human diseases, presents major challenges for treatment and preventing the spread of infection. Without more effective diagnostic tools than what exists today, antimicrobial resistance (AMR) will continue to increase, and treatment options will be increasingly limited, with the establishment of so-called multi-resistant “superbugs”, e.g., Extended Spectrum β-Lactamase (ESBL) and Carbapenem-Resistant Enterobacteriaceae (CRE). The World Health Organization (WHO) has predicted the advent of the post-antibiotic era, facing infections for which no antibiotic treatment will be available (Reardon, 2014) With this prognosis, there is an increasingly critical need to develop new, rapid and reliable methodologies for comprehensive diagnostics of infectious microorganisms and associated virulence and antimicrobial resistance (AMR), to guide more appropriate treatments of infections, reduce the risk of AMR development, prevent mortality and reduce costs associated with treatment and infection control.

The recent evolution of mass spectrometers, with high sensitivity, accuracy and resolution, in conjunction with improved chromatographic separation techniques, enables detection of almost the entire expressed proteome of a microorganism (Armengaud, 2013). A great advantage of the proteotyping approach is that, whereas other traditionally used methods in clinical microbiology diagnostics rely completely on a successful isolation of a pure culture (including MALDI-TOF MS), proteotyping is able to identify tens of thousands of peptides, all potential markers for species, strain, resistance and virulence traits, from the same sample in just one analysis. Hence, proteotyping can identify several different species (or even strains) in a patient sample with a co-infection of bacteria/fungi (Karlsson et al., 2012; Karlsson et al., 2015; Karlsson et al., 2018). The growing amount of genome sequence data enables accurate detection of a growing number and diversity of microorganisms, as well as deeper understanding of traits such as virulence and antimicrobial resistance (AMR). With such analytical means, it is feasible to determine directly, within a clinical sample, the species identity, the sub-species strain type and factors expressing virulence and AMR.

In further studies, peptide biomarkers for blood infectious bacteria or fungi, even on different taxonomical levels, will be investigated. The approach of exploiting those biomarkers as a rapid, accurate and sensitive alternative to traditional, often culture-based, protocols would also need to be investigated. The proteotyping workflow in this paper was applied to demonstrate the feasibility of using peptide biomarkers to detect bacteria and fungi in blood samples, using culture-independent tandem mass spectrometry analyses. Attention was given to optimizing the workflow to reduce cells and proteins of host origin, as well as assessing the sensitivity and accuracy compared to the commonly used MALDI-TOF MS-based identification. At this stage, less attention has been given to the time and cost of the analysis, using the proteotyping workflow. To transfer the peptide biomarker candidates into a clinical setting, especially the cost per sample would need to be specifically addressed, as well as the time required from sample preparation to analysis result. The sample preparation step and the digestion step, to produce the biomarker peptides, are steps in the workflow where there is plenty of potential for reducing the processing time. Indeed, the proteotyping workflow has been shown to be markedly reduced, in some settings, to only 30 minutes (Hayoun et al., 2019). Further optimization and time-saving may include targeted LCMS approaches using already existing triple quadrupole MS instrumentation in the clinic. Furthermore, alternative strategies to reduce the cost and time per sample may include utilization of the unique amino acid sequences of the biomarker peptides found by shotgun proteotyping, as the biomarker information also may hold potential to be transferred into other diagnostics approaches, such as ELISA-based assays or, as previously shown, MALDI-TOF MS (Mery et al., 2016). A key benefit of using proteomics-based methods compared to methods detecting genetic material is the information regarding expression. Further studies will focus on markers for antibiotic resistance and virulence, ideally information regarding species and strain identification will be provided at the same time as information regarding expression of resistance and virulence traits in one single direct analysis of a clinical sample, without any culturing (Charretier et al., 2015; Karlsson et al., 2015).

## Data Availability Statement

The datasets presented in this study can be found in online repositories. The names of the repository/repositories and accession number(s) can be found below: ProteomeXchange *via* the PRIDE database PXD023033.

## Ethics Statement

Ethical review and approval was not required for the study on human participants in accordance with the local legislation and institutional requirements. Written informed consent for participation was not required for this study in accordance with the national legislation and the institutional requirements.

## Author Contributions

RK, AKa, AT, EM, and NKo designed research. AKu, BP-I, TT, NKa, AT, and JF performed research. RK, AKa, Y-KY, GA, and AO contributed new reagents/analytic tools. RK, AT, BP-I, FS-S, DJ-L, JF, AK, Y-KY, GA, and AO analyzed data. All authors contributed to the article and approved the submitted version.

## Funding

RK, FS-S, DJ-L, and EM acknowledge support from the European Commission 7th Framework Programme: “Tailored-Treatment”, EU Grant Agreement No.: HEALTH-F3-602860-2013. Swedish Västra Götaland regional funding, project nos. ALFGBG-437221 supported RK, FS-S, EM, and ALFGBG-720761 supported RK, FS-S and EM. The Swedish Västra Götaland Region, FoU grant number VGFOUREG-665141 and Lab Medicine Project number 51060-6258 supported RK and EM. FS-S, DJ-L, and EM acknowledge support from the Swedish Västra Götaland Region, Lab Medicine Project number 51060-6268. FS-S and DJ-L were supported by stipends for Basic and Advanced Research from the Culture Collection of the University of Gothenburg (CCUG), through the Institute of Biomedicine, Sahlgrenska Academy, University of Gothenburg.

## Conflict of Interest

Authors AKa and RK are affiliated to a company, Nanoxis Consulting AB. The Company did not have influence on the collection, analysis, or interpretation of data, the writing of the paper, or the decision to submit for publication.

The remaining authors declare that the research was conducted in the absence of any commercial or financial relationships that could be construed as a potential conflict of interest.

## Publisher’s Note

All claims expressed in this article are solely those of the authors and do not necessarily represent those of their affiliated organizations, or those of the publisher, the editors and the reviewers. Any product that may be evaluated in this article, or claim that may be made by its manufacturer, is not guaranteed or endorsed by the publisher.

## Abbreviations

AMR, Antimicrobial resistance; BSI, Bloodstream infection; CCUG, Culture Collection University of Gothenburg; CHCA, α-cyano-4-hydroxycinnamic acid; CRE, Carbapenem-Resistant Enterobacteriaceae; ESBL, Extended Spectrum β-Lactamase ; FISH, fluorescence *in-situ* hybridization; IVD *In Vitro* Diagnostics ; LC-MS/MS, liquid chromatography tandem mass spectrometry; MALDI-TOF, matrix assisted laser desorption/ionization time of flight; MS, mass spectrometry ; MiCld, Microorganism Classification and Identification; mmu, millimass units; OD, Optical Density; ON, overnight; PRM, parallel reaction monitoring; RUO, research use only; SDC, sodium deoxycholate; TCUP, Typing and Characterisation Using Proteomics; VIR, virulence; WHO, World Health Organization.
